# Validation List no. 219. Valid publication of new names and new combinations effectively published outside the IJSEM

**DOI:** 10.1099/ijsem.0.006452

**Published:** 2024-10-03

**Authors:** Aharon Oren, Markus Göker

**Affiliations:** 1 The Institute of Life Sciences, The Hebrew University of Jerusalem, The Edmond J. Safra Campus, 9190401 Jerusalem, Israel; 2 Leibniz Institute DSMZ – German Collection of Microorganisms and Cell Cultures, Inhoffenstrasse 7B, 38124 Braunschweig, Germany

The purpose of this list is to publish in the *International Journal of Systematic and Evolutionary Microbiology* (IJSEM) those new names and new combinations that were effectively published outside the IJSEM as set forth in Rule 27 of the International Code of Nomenclature of Prokaryotes (2022 Revision) [1]. Authors and other individuals wishing to have new names and/or combinations included in future lists should send an electronic copy of the published paper to the IJSEM Editorial Office for confirmation that all of the other requirements for valid publication have been met. It is also a requirement of IJSEM and the International Code of Nomenclature of Prokaryotes (ICNP) that authors of new species, new subspecies and new combinations provide evidence that types are deposited in two recognized culture collections in two different countries. It should be noted that the date of valid publication of these new names and combinations is the date of publication of this list, not the date of the original publication of the names and combinations. The authors of the new names and combinations are as given below.

Inclusion of a name on these lists validates the publication of the name and thereby makes it available in the nomenclature of prokaryotes. Inclusion of a name on these lists is part of the requirements of valid publication of an effectively published name. Indeed, some of these names may, in time, be considered later synonyms, or it may be proposed to transfer the organisms to another genus, thus necessitating the creation of a new combination. Conversely, it may be proposed to transfer an organism back to an earlier genus and to regard the basonym or an earlier new combination as the correct name instead of a more recent new combination. The ICNP does not decide on such taxonomic opinions.

**Table:** 

Name/authors	Proposed as	Nomenclatural type^1^	Priority^2^	Reference
*Actinacidiphila acidipaludis* (Nammali *et al*. 2022) Komaki *et al*. 2024, 694	comb. nov. [basonym: *Streptomyces acidipaludis* Nammali *et al*. 2022]	PLK6-54 (=NBRC 114297=TBRC 11250)	57	[2]
*Agromyces chromiiresistens* Liu *et al*. 2023, 11^3^	sp. nov.	H3Y2-19a (=CGMCC 1.61332=JCM 36199)	53	[3]
*Alicyclobacillus acidocaldarius* subsp. *rittmannii* Nicolaus *et al*. 1998, 140^4^	subsp. nov.	MR1 (=CCUG 59834=DSM 11297)^5^	11	[4]
*Aliigemmobacter* Huang *et al*. 2024, 17^3^	gen. nov.	*Aliigemmobacter aestuarii*	36	[5]
*Aliigemmobacter aestuarii* (Hameed *et al*. 2020) Huang *et al*. 2024, 18^3^	comb. nov. [basonym: *Gemmobacter aestuarii* Hameed *et al*. 2020]	CC-PW-75 (=BCRC 80759=JCM 19754)	36	[5]
*Aliirhizobium* corrig. Ma *et al*. 2023, 9^3,6^	gen. nov.	*Aliirhizobium cellulosilyticum* corrig.	48	[6]
*Aliirhizobium cellulosilyticum* corrig. (García-Fraile *et al*. 2007) Ma *et al*. 2023, 12^3,7^	comb. nov. [basonym: *Rhizobium cellulosilyticum* García-Fraile *et al*. 2007]	ALA10B2 (=DSM 18291=LMG 23642)^8^	48	[6]
*Aliirhizobium smilacinae* corrig. (Zhang *et al*. 2015) Ma *et al*. 2023, 12^3^	comb. nov. [basonym: *Rhizobium smilacinae* Zhang *et al*. 2015]	PTYR-5 (=DSM 100675=LMG 27604)^9^	48	[6]
*Aliiroseovarius salicola* Yoon 2024, 5^3^	sp. nov.	KMU-50 (=KCCM 90480=NBRC 115482)	51	[7]
*Aliitabrizicola* Huang *et al*. 2024, 17^3^	gen. nov.	*Aliitabrizicola rongguiensis*	36	[5]
*Aliitabrizicola rongguiensis* (Xu *et al*. 2022) Huang *et al*. 2024, 17^3^	comb. nov. [basonym: *Tabrizicola rongguiensis* Xu *et al*. 2022]	J26 (=GDMCC 1.2843=KCTC 92112)	36	[5]
*Altererythrobacter litoralis* Liu *et al*. 2024, 8^3^	sp. nov.	1XM1-14 (=GDMCC 1.2383=KCTC 82612)	59	[8]
*Amycolatopsis cynarae* Deng *et al*. 2024, 6^3^	sp. nov.	HUAS 11–8 (=JCM 35980=MCCC 1K08337)	40	[9]
*Aquipseudomonas* Rudra and Gupta 2024, 20^3^	gen. nov.	*Aquipseudomonas alcaligenes* ^10^	37	[10]
*Aquipseudomonas alcaligenes* (Monias 1928) Rudra and Gupta 2024, 21^3^	comb. nov. [basonym: *Pseudomonas alcaligenes* Monias 1928 (Approved Lists 1980)]^10^	1577 (=DSM 50342=NCTC 10367)^11^	37	[10]
*Aquipseudomonas campi* (Timsy *et al*. 2021) Rudra and Gupta 2024, 21^3^	comb. nov. [basonym: *Pseudomonas campi* Timsy *et al*. 2021]	S1-A32-2 (=DSM 110222=LMG 31521)	37	[10]
*Aquipseudomonas guryensis* (Kim *et al*. 2021) Rudra and Gupta 2024, 21^3^	comb. nov. [basonym: *Pseudomonas guryensis* Kim *et al*. 2021]	SR9 (=JCM 34509=KCTC 82228)	37	[10]
*Aquipseudomonas ullengensis* (Kim *et al*. 2021) Rudra and Gupta 2024, 21^3^	comb. nov. [basonym: *Pseudomonas ullengensis* Kim *et al*. 2021]	UL070 (=JCM 34510=KCTC 82229)	37	[10]
*Aureispira anguillae* Yuasa *et al*. 2024, 8^3^	sp. nov.	EL160426 (=ATCC TSD-286=JCM 35024)^12^	13	[11]
*Azotosporobacter* Xie *et al*. 2024, 7^3^	gen. nov.	*Azotosporobacter soli*	64	[12]
*Azotosporobacter soli* Xie *et al*. 2024, 8^3^	sp. nov.	SG130 (=GDMCC 1.3312=JCM 35641)	64	[12]
*Bosea rubneri* Stoll *et al*. 2024, 6^3^	sp. nov.	ZW T0_25 (=DSM 116094=LMG 33093)^13^	70	[13]
*Bradyrhizobium ontarionense* Bromfield and Cloutier 2024, 9^3^	sp. nov.	A19 (=HAMBI 3761=LMG 32638)	26	[14]
*Cellulomonas endometrii* Abou Chacra *et al*. 2023, 8^3^	sp. nov.	Marseille-Q7820 (=CECT 30716=CSUR Q7820)	2	[15]
*Chryseomonas oryzihabitans* (Kodama *et al*. 1985) Rudra and Gupta 2024, 24^3^	comb. nov. [basonym: *Pseudomonas oryzihabitans* Kodama *et al*. 1985]	L-1 (=ATCC 43272=CCUG 12540)^14^	37	[10]
*Chryseomonas psychrotolerans* (Hauser *et al*. 2004) Rudra and Gupta 2024, 24^3^	comb. nov. [basonym: *Pseudomonas psychrotolerans* Hauser *et al*. 2004]	C36 (=DSM 15758=LMG 21977)^15^	37	[10]
*Chryseomonas zeshuii* (Feng *et al*. 2012) Rudra and Gupta 2024, 24^3^	comb. nov. [basonym: *Pseudomonas zeshuii* Feng *et al*. 2012]	BY; BY-1 (=DSM 27927=NTCC 790297)^16^	37	[10]
*Companilactobacillus muriivasis* Zhang *et al*. 2024, 5^3^	sp. nov.	419–1.2 (=CCTCC AB 2022413=JCM 35891)	66	[16]
*Corynebacterium ihumii* Padmanabhan *et al*. 2014, 1139	sp. nov.	GD7 (=CSUR P902=DSM 45751)	20	[17]
*Ectopseudomonas* Rudra and Gupta 2024, 24^3^	gen. nov.	*Ectopseudomonas oleovorans*	37	[10]
*Ectopseudomonas alcaliphila* (Yumoto *et al*. 2001) Rudra and Gupta 2024, 21^3^	comb. nov. [basonym: *Pseudomonas alcaliphila* Yumoto *et al*. 2001]	AL15-21 (=DSM 17744=JCM 10630)^17^	37	[10]
*Ectopseudomonas chengduensis* (Tao *et al*. 2014) Rudra and Gupta 2024, 21^3^	comb. nov. [basonym: *Pseudomonas chengduensis* Tao *et al*. 2014]	MBR (=BCRC 80835=DSM 26382)^18^	37	[10]
*Ectopseudomonas composti* (Gibello *et al*. 2011) Rudra and Gupta 2024, 21^3^	comb. nov. [basonym: *Pseudomonas composti* Gibello *et al*. 2011]	C2 (=CCUG 59231=DSM 25648)^19^	37	[10]
*Ectopseudomonas guguanensis* (Liu *et al*. 2013) Rudra and Gupta 2024, 21^3^	comb. nov. [basonym: *Pseudomonas guguanensis* Liu *et al*. 2013]	CC-G9A (=BCRC 80438=JCM 18416)	37	[10]
*Ectopseudomonas hydrolytica* (Zhou *et al*. 2020) Rudra and Gupta 2024, 21^3^	comb. nov. [basonym: *Pseudomonas hydrolytica* Zhou *et al*. 2020]	DSWY01 (=CCTCC AB 2018053=DSM 106702)	37	[10]
*Ectopseudomonas khazarica* (Tarhriz *et al*. 2020) Rudra and Gupta 2024, 22^3^	comb. nov. [basonym: *Pseudomonas khazarica* Tarhriz *et al*. 2020]	TBZ2 (=LMG 29674=KCTC 52410)	37	[10]
*Ectopseudomonas mendocina* (Palleroni 1970) Rudra and Gupta 2024, 22^3^	comb. nov. [basonym: *Pseudomonas mendocina* Palleroni 1970 (Approved Lists 1980)]^10^	CH 50 (=ATCC 25411=DSM 50017)^20^	37	[10]
*Ectopseudomonas oleovorans* (Lee and Chandler 1941) Rudra and Gupta 2024, 21^3^	comb. nov. [basonym: *Pseudomonas oleovorans* Lee and Chandler 1941 (Approved Lists 1980)]	DSM 1045=JCM 11598^21^	37	[10]
*Ectopseudomonas pseudoalcaligenes* (Stanier 1966) Rudra and Gupta 2024, 22^3^	comb. nov. [basonym: *Pseudomonas pseudoalcaligenes* Stanier 1966 (Approved Lists 1980)]	LMG 1225=NBRC 14167^22^	37	[10]
*Ectopseudomonas toyotomiensis* (Hirota *et al*. 2011) Rudra and Gupta 2024, 22^3^	comb. nov. [basonym: *Pseudomonas toyotomiensis* Hirota *et al*. 2011]	HT-3 (=DSM 26169=JCM 15604)^23^	37	[10]
*Ensifer collicola* Jang *et al*. 2017, 523	sp. nov.	Mol12 (=JCM 31049=KCTC 42816)	23	[18]
*Falsirhodobacter flavus* (Liu *et al*. 2023) Huang *et al*. 2024, 17^3^	comb. nov. [basonym: *Cereibacter flavus* Liu *et al*. 2023]	SYSU M79828 (=GDMCC 1.3803=KCTC 92893)	36	[5]
*Falsirhodobacter xinxiangensis* (Han *et al*. 2024) Huang *et al*. 2024, 17^3^	comb. nov. [basonym: *Rhodobacter xinxiangensis* Han *et al*. 2024]	TJ48 (=CCTCC AB 2019120=KCTC 72510)	36	[5]
*Falsiroseicyclus* Huang *et al*. 2024, 16^3^	gen. nov.	*Falsiroseicyclus tamaricis*	36	[5]
*Falsiroseicyclus tamaricis* (Gai *et al*. 2021) Huang *et al*. 2024, 16^3^	comb. nov. [basonym: *Pseudoroseicyclus tamaricis* Gai *et al*. 2021]	CLL3-39 (=KCTC 72665=MCCC 1A14815)	36	[5]
*Ferdinandcohnia quinoae* Saati-Santamaría *et al*. 2024, 177	sp. nov.^24^	SECRCQ15 (=CECT 30513=LMG 32511)	1	[19]
*Flavobacterium cupriresistens* Saticioglu *et al*. 2024, 3^3^	sp. nov.	F-323 (=JCM 34200=KCTC 82260)	58	[20]
*Flavobacterium flavipigmentatum* Saticioglu *et al*. 2024, 3^3^	sp. nov.	F-70 (=JCM 34198=KCTC 82258)	58	[20]
*Flavobacterium lipolyticum* Saticioglu *et al*. 2024, 3^3^	sp. nov.	F126 (=JCM 34199=KCTC 82259)	58	[20]
*Flavobacterium pisciphilum* Saticioglu *et al*. 2024, 3^3^	sp. nov.	F65 (=JCM 34197=KCTC 82257)	58	[20]
*Flavobacterium piscisymbiosum* Saticioglu *et al*. 2024, 3^3^	sp. nov.	F30 (=JCM 34194=KCTC 82254)	58	[20]
*Fodinisporobacter* Jiang *et al*. 2024, 10^3^	gen. nov.	*Fodinisporobacter ferrooxydans*	54	[21]
*Fodinisporobacter ferrooxydans* Jiang *et al*. 2024, 10^3^	sp. nov.	MYW30-H2 (=CGMCC 1.17422=KCTC 43278)	54	[21]
*Formicincola* Chua *et al*. 2022, 1002	gen. nov.	*Formicincola oecophyllae*	62	[22]
*Formicincola oecophyllae* Chua *et al*. 2022, 1002	sp. nov.	F3b2 (=DSM 106908=LMG 30590)^25^	62	[22]
*Fructobacillus evanidus* Botero *et al*. 2024, 5^3^	sp. nov.	LMG 32999 (=CECT 30959)	8	[23]
*Geopseudomonas* Rudra and Gupta 2024, 25^3^	gen. nov.	*Geopseudomonas sagittaria*	37	[10]
*Geopseudomonas aromaticivorans* (Banerjee *et al*. 2023) Rudra and Gupta 2024, 22^3^	comb. nov. [basonym: *Pseudomonas aromaticivorans* Banerjee *et al*. 2023]	MAP12 (=LMG 32466=NCAIM B.02668)	37	[10]
*Geopseudomonas guangdongensis* (Yang *et al*. 2013) Rudra and Gupta 2024, 22^3^	comb. nov. [basonym: *Pseudomonas guangdongensis* Yang *et al*. 2013]	SgZ-6 (=CCUG 72322=DSM 100318)^26^	37	[10]
*Geopseudomonas sagittaria* (Lin *et al*. 2013) Rudra and Gupta 2024, 22^3^	comb. nov. [basonym: *Pseudomonas sagittaria* Lin *et al*. 2013]	CC-OPY-1 (=DSM 27945=JCM 18195)^27^	37	[10]
*Geothrix campi* Han *et al*. 2024, 7^3^	sp. nov.	SG10 (=GDMCC 1.3406=JCM 39331)	63	[24]
*Geothrix mesophila* Han *et al*. 2024, 8^3,28^	sp. nov.	SG198 (=GDMCC 1.3309=KCTC 25635)^29^	63	[24]
*Gordoniibacillus* corrig. Kudryashova *et al*. 2024, 277^30^	gen. nov.	*Gordoniibacillus kamchatkensis* corrig.	42	[25]
*Gordoniibacillus kamchatkensis* corrig. Kudryashova *et al*. 2024, 277	sp. nov.	V-9 (=DSM 26726=VKM B-2647)	42	[25]
*Halocynthiibacter styelae* (Kim *et al*. 2021) Huang *et al*. 2024, 16^3^	comb. nov. [basonym: *Paenihalocynthiibacter styelae* Kim *et al*. 2021]	MYP1-1 (=KCTC 82143=NBRC 114355)	36	[5]
*Halomarina halobia* Cheng *et al*. 2024, 7^3^	sp. nov.	PSR21 (=CGMCC 1.17027=JCM 34147)	61	[26]
*Halomarina litorea* Cheng *et al*. 2024, 6^3^	sp. nov.	BCD28 (=CGMCC 1.18776=JCM 34908)	61	[26]
*Halomarina ordinaria* Cheng *et al*. 2024, 7^3^	sp. nov.	PSRA2 (=CGMCC 1.17214=JCM 34148)	61	[26]
*Halomarina pelagica* Cheng *et al*. 2024, 6^3^	sp. nov.	BND7 (=CGMCC 1.18778=JCM 34910)	61	[26]
*Hyalangium rubrum* corrig. Zang *et al*. 2024, 7^3,31^	sp. nov.	s54d21 (=GDMCC 1.1945=JCM 39263)	45	[27]
*Infirmifilum uzonense* (Toshchakov *et al*. 2016) Zayulina *et al*. 2021, 12^3^	comb. nov. [basonym: *Thermofilum uzonense* Toshchakov *et al*. 2016]^32^	1807–2 (=DSM 28062=JCM 19810)	19	[28]
*Jejuia spongiicola* Kim *et al*. 2022, 272	sp. nov.	2012CJ34-3 (=KACC 22642=LMG 32583)	24	[29]
*Lacticaseibacillus salsurivasis* Zhang *et al*. 2024, 3^3^	sp. nov.	74–4 (=CCTCC AB 2022414=JCM 35890)	66	[16]
*Lysinibacillus piscis* Kanbe *et al*. 2024, 7^3^	sp. nov.	KH24 (=JCM 36611=KCTC 43676)	33	[30]
*Maribacter halichondriae* Steiner *et al*. 2024, 13^3^	sp. nov.	Hal144 (=DSM 114563=LMG 32744)	69	[31]
*Meridianimarinicoccus aquatilis* (Sun *et al*. 2020) Huang *et al*. 2024, 17^3^	comb. nov. [basonym: *Fluviibacterium aquatile* Sun *et al*. 2020]	SM1902 (=KCTC 72045=MCCC 1K03596)^33^	36	[5]
*Meridianimarinicoccus zhengii* (Zhu *et al*. 2019) Huang *et al*. 2024, 16^3^	comb. nov. [basonym: *Phycocomes zhengii* Zhu *et al*. 2019]	LMIT002 (=CICC 24357=KCTC 62390)	36	[5]
*Metapseudomonas* Rudra and Gupta 2024, 25^3^	gen. nov.	*Metapseudomonas resinovorans*	37	[10]
*Metapseudomonas boanensis* (Nicklasson *et al*. 2022) Rudra and Gupta 2024, 23^3^	comb. nov. [basonym: *Pseudomonas boanensis* Nicklasson *et al*. 2022]	DB1 (=CCUG 62977=CECT 30359)	37	[10]
*Metapseudomonas furukawaii* (Kimura *et al*. 2018) Rudra and Gupta 2024, 23^3^	comb. nov. [basonym: *Pseudomonas furukawaii* Kimura *et al*. 2018]	KF707 (=DSM 10086=NBRC 110670)	37	[10]
*Metapseudomonas lalkuanensis* (Thorat *et al*. 2020) Rudra and Gupta 2024, 23^3^	comb. nov. [basonym: *Pseudomonas lalkuanensis* Thorat *et al*. 2020]	PE08 (=CCUG 73691=KCTC 72454)^34^	37	[10]
*Metapseudomonas otitidis* (Clark *et al*. 2006) Rudra and Gupta 2024, 23^3^	comb. nov. [basonym: *Pseudomonas otitidis* Clark *et al*. 2006]^10^	MCC 10330 (=ATCC BAA-1130=DSM 17224)	37	[10]
*Metapseudomonas resinovorans* (Delaporte *et al*. 1961) Rudra and Gupta 2024, 23^3^	comb. nov. [basonym: *Pseudomonas resinovorans* Delaporte *et al*. 1961 (Approved Lists 1980)]	DSM 21078=LMG 2274^35^	37	[10]
*Methylobacter svalbardensis* Patil *et al*. 2024, 16^3,36^	sp. nov.	LS7-T4A (=DSM 114308=JCM 39463)^37^	49	[32]
*Microbacterium dauci* Zheng *et al*. 2024, 5^3,38^	sp. nov.	LX3-4 (=CCTCC AB 2023103=LMG 33159)	12	[33]
*Micromonospora robiginosa* Back *et al*. 2023, 1^3,39^	nom. nov. [basonym: *Micromonospora ferruginea* Back *et al.* 2021]	28ISP2-46 (=DSM 111791=NCIMB 15402)	50	[34]
*Mobiluncus massiliensis* Abou Chacra *et al*. 2024, 11^3^	sp. nov.	Marseille-Q7826 (=CECT 30727=CSUR Q7826)	17	[35]
*Neglectibacter* Zgheib *et al*. 2022, 7^3^	gen. nov.	*Neglectibacter timonensis*	35	[36]
*Neglectibacter timonensis* Zgheib *et al*. 2022, 8^3^	sp. nov.	Marseille-P2265 (=CSUR P2265=DSM 102082)	35	[36]
*Neogemmobacter* Huang *et al*. 2024, 18^3^	gen. nov.	*Neogemmobacter tilapiae*	36	[5]
*Neogemmobacter tilapiae* (Sheu *et al*. 2013) Huang *et al*. 2024, 18^3^	comb. nov. [basonym: *Gemmobacter tilapiae* Sheu *et al*. 2013]	Ruye-53 (=BCRC 80261=KCTC 23310)	36	[5]
*Neptunicoccus cionae* (Wang *et al*. 2017) Huang *et al*. 2024, 16^3^	comb. nov. [basonym: *Amylibacter cionae* Wang *et al*. 2017]	H-12 (=CGMCC 1.15880=KCTC 52581)	36	[5]
*Nocardioides turkmenicus* Saygin *et al*. 2024, 11^3^	sp. nov.	KC13 (=CGMCC 4.7619=JCM 33525)	46	[37]
*Oceaniglobus roseus* (Wang *et al*. 2018) Huang *et al*. 2024, 17^3^	comb. nov. [basonym: *Kandeliimicrobium roseum* Wang *et al*. 2018]	XY-R6 (=KCTC 52266=MCCC 1K01498)^40^	36	[5]
*Ostreiculturibacter* Huang *et al*. 2024, 15^3^	gen. nov.	*Ostreiculturibacter nitratireducens*	36	[5]
*Ostreiculturibacter nitratireducens* Huang *et al*. 2024, 15^3^	sp. nov.	FR2A1 (=KCTC 8317=MCCC 1K08809)	36	[5]
*Paenirhodobacter ferrireducens* (Yang *et al*. 2018) Huang *et al*. 2024, 19^3^	comb. nov. [basonym: *Sinirhodobacter ferrireducens* corrig. Yang *et al*. 2018]	SgZ-3 (=CCTCC AB 2012026=KACC 16603)	36	[5]
*Paenirhodobacter hankyongi* (Lee *et al*. 2020) Huang *et al*. 2024, 19^3^	comb. nov. [basonym: *Sinirhodobacter hankyongi* Lee *et al*. 2020]	BO-81 (=KACC 19677=LMG 30808)	36	[5]
*Paenirhodobacter huangdaonensis* (Xi *et al*. 2019) Huang *et al*. 2024, 19^3^	comb. nov. [basonym: *Sinirhodobacter huangdaonensis* corrig. Xi *et al*. 2019]	L3 (=CGMCC 1.12963=KCTC 42823)	36	[5]
*Paenirhodobacter populi* (Xu *et al*. 2019) Huang *et al*. 2024, 19^3^	comb. nov. [basonym: *Sinirhodobacter populi* corrig. Xu *et al*. 2019]	sk2b1 (=CFCC 14580=KCTC 52802)	36	[5]
*Paracerasibacillus* Miliotis *et al*. 2024, 8^3^	gen. nov.	*Paracerasibacillus soli*	7	[38]
*Paracerasibacillus soli* (Kämpfer *et al*. 2011) Miliotis *et al*. 2024, 8^3^	comb. nov. [basonym: *Virgibacillus soli* Kämpfer *et al*. 2011]	CC-YMP-6 (=CCM 7714=DSM 22952)	7	[38]
*Paraflavitalea pollutisoli* Liu *et al*. 2024, 10^3^	sp. nov.	H1-2-19X (=CGMCC 1.61321=JCM 36460)	55	[39]
*Paragemmobacter* Huang *et al*. 2024, 18^3^	gen. nov.	*Paragemmobacter straminiformis*	36	[5]
*Paragemmobacter amnigenus* (Chen *et al*. 2021) Huang *et al*. 2024, 18^3^	comb. nov. [basonym: *Rhodobacter amnigenus* Chen *et al*. 2021]	HSP-20 (=BCRC 81193=LMG 31334)	36	[5]
*Paragemmobacter aquarius* (Baek *et al*. 2020) Huang *et al*. 2024, 18^3^	comb. nov. [basonym: *Gemmobacter aquarius* Baek *et al*. 2020]	HYN0069 (=KACC 19488=NBRC 113115)	36	[5]
*Paragemmobacter kunshanensis* (Liu *et al*. 2024) Huang *et al*. 2024, 18^3^	comb. nov. [basonym: *Rhodobacter kunshanensis* Liu *et al*. 2024]	HX-7–19 (=CCTCC AB 2020148=KCTC 72471)	36	[5]
*Paragemmobacter ruber* (Chen *et al*. 2021) Huang *et al*. 2024, 18^3^	comb. nov. [basonym: *Rhodobacter ruber* Chen *et al*. 2021]	CCP-1 (=BCRC 81189=LMG 31335)	36	[5]
*Paragemmobacter straminiformis* (Kang *et al*. 2017) Huang *et al*. 2024, 18^3^	comb. nov. [basonym: *Gemmobacter straminiformis* Kang *et al*. 2017]	CAM-8 (=JCM 31905=KACC 19224)	36	[5]
*Paramylibacter* Huang *et al*. 2024, 16^3^	gen. nov.	*Paramylibacter ulvae*	36	[5]
*Paramylibacter kogurei* (Wong *et al*. 2018) Huang *et al*. 2024, 16^3^	comb. nov. [basonym: *Amylibacter kogurei* Wong *et al*. 2018]	4G11 (=KCTC 52506=NBRC 112428)^41^	36	[5]
*Paramylibacter ulvae* (Nedashkovskaya *et al*. 2016) Huang *et al*. 2024, 16^3^	comb. nov. [basonym: *Amylibacter ulvae* Nedashkovskaya *et al*. 2016]	6Alg 255 (=KCTC 32465=KMM 6515)	36	[5]
*Parasedimentitalea huanghaiensis* (Xu *et al*. 2021) Huang *et al*. 2024, 16^3^	comb. nov. [basonym: *Zongyanglinia huanghaiensis* Xu *et al*. 2021]	CY05 (=KCTC 62200=MCCC 1K04409)	36	[5]
*Parasedimentitalea marina* (Choi *et al*. 2019) Huang *et al*. 2024, 16^3^	comb. nov. [basonym: *Pelagicola marinus* Choi *et al*. 2019]	DSW4-44 (=JCM 33637=KCTC 62762)^42^	36	[5]
*Parasulfitobacter* Xu *et al*. 2024, 11^3,43^	gen. nov.	*Parasulfitobacter algicola*	29	[40]
*Parasulfitobacter algicola* (Wang *et al*. 2022) Xu *et al*. 2024, 11^3^	comb. nov. [basonym: *Sulfitobacter algicola* Wang *et al*. 2022]	1151 (=KCTC 72513=MCCC 1H00384)^44^	29	[40]
*Parendozoicomonas callyspongiae* Kim *et al*. 2024, 7^3,45^	sp. nov.	2012CJ34-2 (=KACC 22641=LMG 32581)	9	[41]
*Peptoniphilus genitalis* Abou Chacra *et al*. 2024, 10^3^	sp. nov.	Marseille-Q7072 (=CECT 30604=CSUR Q7072)	17	[35]
*Phytopseudomonas* Rudra and Gupta 2024, 25^3^	gen. nov.	*Phytopseudomonas straminea*	37	[10]
*Phytopseudomonas argentinensis* (Peix *et al*. 2005) Rudra and Gupta 2024, 23^3^	comb. nov. [basonym: *Pseudomonas argentinensis* Peix *et al*. 2005]	CH01 (=DSM 17259=LMG 22563)^46^	37	[10]
*Phytopseudomonas daroniae* (Bueno-Gonzalez *et al*. 2019) Rudra and Gupta 2024, 23^3^	comb. nov. [basonym: *Pseudomonas daroniae* Bueno-Gonzalez *et al*. 2019]	FRB228 (=LMG 31088=NCPPB 4672)	37	[10]
*Phytopseudomonas dryadis* (Bueno-Gonzalez *et al*. 2019) Rudra and Gupta 2024, 23^3^	comb. nov. [basonym: *Pseudomonas dryadis* Bueno-Gonzalez *et al*. 2019]	FRB230 (=LMG 31087=NCPPB 4673)	37	[10]
*Phytopseudomonas flavescens* (Hildebrand *et al*. 1994) Rudra and Gupta 2024, 23^3^	comb. nov. [basonym: *Pseudomonas flavescens* Hildebrand *et al*. 1994]	B62 (=DSM 12071=LMG 18387^47^	37	[10]
*Phytopseudomonas punonensis* (Ramos *et al*. 2013) Rudra and Gupta 2024, 23^3^	comb. nov. [basonym: *Pseudomonas punonensis* Ramos *et al*. 2013]	LMT03 (=DSM 27507=LMG 26839)^48^	37	[10]
*Phytopseudomonas seleniipraecipitans* (Hunter and Manter 2011) Rudra and Gupta 2024, 23^3^	comb. nov. [basonym: *Pseudomonas seleniipraecipitans* corrig. Hunter and Manter 2011]	CA5 (=DSM 25106=LMG 25475)^49^	37	[10]
*Phytopseudomonas straminea* (Iizuka and Komagata 1963) Rudra and Gupta 2024, 23^3^	comb. nov. [basonym: *Pseudomonas straminea* corrig. Iizuka and Komagata 1963 (Approved Lists 1980)]	CCUG 12539=DSM 17727^50^	37	[10]
*Pollutibacter* Liu *et al*. 2024, 10^3^	gen. nov.	*Pollutibacter soli*	55	[39]
*Pollutibacter soli* Liu *et al*. 2024, 10^3^	sp. nov.	JS81 (=CGMCC 1.61338=JCM 36462)	55	[39]
*Polluticoccus* Liu *et al*. 2024, 10^3^	gen. nov.	*Polluticoccus soli*	55	[39]
*Polluticoccus soli* Liu *et al*. 2024, 10^3^	sp. nov.	JY13-12 (=CGMCC 1.61341=JCM 36463)	55	[39]
*Pontiella agarivorans* Liu *et al*. 2024, 17^3^	sp. nov.	NLcol2 (=DSM 113125=MCCC 1K08672)	10	[42]
*Pontivivens marinum* (Chernikova *et al*. 2017) Huang *et al*. 2024, 15^3^	comb. nov. [basonym: *Monaibacterium marinum* Chernikova *et al*. 2017]	C7 (=DSM 100241=LMG 28800)	36	[5]
*Pontivivens nitratireducens* (Liang *et al*. 2022) Huang *et al*. 2024, 15^3^	comb. nov. [basonym: *Pontibrevibacter nitratireducens* Liang *et al*. 2022]	h42 (=CGMCC 1.17849=KCTC 72875=MCCC 1K04735)	36	[5]
*Porphyromonas vaginalis* Abou Chacra *et al*. 2024, 10^3^	sp. nov.	Marseille-P5150 (=CECT 30262=CSUR P5150)	3	[43]
*Pseudogemmobacter blasticus* (Eckersley and Dow 1981) Huang *et al*. 2024, 18^3^	comb. nov. [basonym: *Rhodopseudomonas blastica* Eckersley and Dow 1981]	NCIB 11576 (=ATCC 33485=NBRC 16437)	36	[5]
*Pseudomaricurvus albidus* (Xue *et al*. 2022) Montecillo *et al*. 2024, 8^3^	comb. nov. [basonym: *Aestuariicella albida* Xue *et al*. 2022]	HHU G3-2 (=JCM 34652=MCCC 1K04224)^51^	31	[44]
*Pseudomaricurvus hydrocarbonicus* (Lo *et al*. 2015) Montecillo *et al*. 2024, 8^3^	comb. nov. [basonym: *Aestuariicella hydrocarbonica* Lo *et al*. 2015]	SM-6 (=JCM 30134=KACC 18121)	31	[44]
*Pseudothioclava nitratireducens* (Liu *et al*. 2017) Huang *et al*. 2024, 18^3^	comb. nov. [basonym: *Defluviimonas nitratireducens* Liu *et al*. 2017]	DL-5–4 (=LMG 29616=MCCC 1A06955)	36	[5]
*Ralstonia condita* Steyaert *et al*. 2024, 12^3^	sp. nov.	LMG 7141 (=CCUG 77163)	68	[45]
*Ralstonia edaphi* corrig. Steyaert *et al*. 2024, 13^3,52^	sp. nov.	LMG 6871 (=CCUG 18841)	68	[45]
*Ralstonia flaminis* Steyaert *et al*. 2024, 12^3^	sp. nov.	LMG 18101 (=CCUG 38754)	68	[45]
*Ralstonia flatus* Steyaert *et al*. 2024, 12^3^	sp. nov.	LMG 32965 (=CCUG 77161)	68	[45]
*Ralstonia holmesii* Steyaert *et al*. 2024, 12^3^	sp. nov.	LMG 32967 (=CCUG 77162)	68	[45]
*Ralstonia psammae* Steyaert *et al*. 2024, 13^3^	sp. nov.	LMG 19083 (=CCUG 77164)	68	[45]
*Ralstonia thomasii* Steyaert *et al*. 2024, 12^3^	sp. nov.	LMG 18095 (=CCUG 38763)	68	[45]
*Raoultella lignicola* Brady *et al*. 2024, 9^3^	sp. nov.	TW_WC1a.1 (=CCUG 77094=LMG 33073)	30	[46]
*Raoultella scottii* corrig. Brady *et al*. 2024, 9^3,53^	sp. nov.	BAC 10 a-01-01 (=CCUG 77096=LMG 33072)	30	[46]
*Rhizobium rhizolycopersici* Thin *et al*. 2021, 835^54^	sp. nov.	DBTS2 (=CICC 24887=JCM 34245)^55^	21	[47]
*Mycoplana rhizolycopersici* (Thin *et al*. 2021) Ma *et al*. 2023, 10^3,56^	comb. nov. [basonym: *Rhizobium rhizolycopersici* Thin *et al*. 2021]	DBTS2 (=CICC 24887=JCM 34245)^57^	22	[6]
*Rhodocytophagaceae* Zhang *et al*. 2023, 9^3,58^	fam. nov.	*Rhodocytophaga*	47	[48]
*Rhodoferax antarcticus* Madigan *et al*. 2000, 275^59^	sp. nov.	ANT.BR (=ATCC 700587=DSM 24876)^60^	14	[49]
*Roseinatronobacter bogoriensis* (Milford *et al*. 2001) Huang *et al*. 2024, 17^3^	comb. nov. [basonym: *Rhodobaca bogoriensis* Milford *et al*. 2001]	LBB1 (=ATCC 700920=DSM 18756)	36	[5]
*Roseinatronobacter domitianus* (Gattoni *et al*. 2024) Huang *et al*. 2024, 17^3^	comb. nov. [basonym: *Roseibaca domitiana* Gattoni *et al*. 2024]^61^	V10 (=CECT 30319=DSM 112951=LMG 32429)	36	[5]
*Roseinatronobacter ekhonensis* (Labrenz *et al*. 2009) Huang *et al*. 2024, 17^3^	comb. nov. [basonym: *Roseibaca ekhonensis* Labrenz *et al*. 2009]	EL-50 (=CECT 7235=DSM 11469)	36	[5]
*Sabulicella* Lin *et al*. 2022, 3^3^	gen. nov.	*Sabulicella rubraurantiaca*	27	[50]
*Sabulicella rubraurantiaca* Lin *et al*. 2022, 4^3^	sp. nov.	SYSU D01096 (=CGMCC 1.8619=KCTC 82268=MCCC 1K04998)	27	[50]
*Saccharopolyspora ipomoeae* Suksaard *et al*. 2024, 7^3^	sp. nov.	TS4A08 (=NBRC 115967=TBRC 17271)	44	[51]
*Salinibacterium metalliresistens* Liu *et al*. 2023, 11^3^	sp. nov.	H3M29-4 (=CGMCC 1.61335=JCM 36200)	53	[3]
*Schauerella* Behrendt *et al*. 2024, 10^3^	gen. nov.	*Schauerella fraxinea*	56	[52]
*Schauerella aestuarii* (Kim *et al*. 2023) Behrendt *et al*. 2024, 10^3^	comb. nov. [basonym: *Achromobacter aestuarii* Kim *et al*. 2023]	KS-M25 (=JCM 33329=KACC 21219)	56	[52]
*Schauerella fraxinea* Behrendt *et al*. 2024, 10^3^	sp. nov.	B3P038 (=DSM 115926=LMG 33092)	56	[52]
*Sedimentitalea arenosa* (Jeong *et al*. 2024) Huang *et al*. 2024, 16^3^	comb. nov. [basonym: *Arenibacterium arenosum* Jeong *et al*. 2024]	CAU1593 (=KCTC 82402=MCCC 1K05671)	36	[5]
*Serpens tuomuerensis* (Xin *et al*. 2009) Rudra and Gupta 2024, 24^3^	comb. nov. [basonym: *Pseudomonas tuomuerensis* Xin *et al*. 2009]	78–123 (=CECT 7766=CGMCC 1.1365)^62^	37	[10]
*Sphaerotilus microaerophilus* Narihara *et al*. 2024, 8^3^	sp. nov.	FB-5 (=JCM 35424=KACC 23146)	38	[53]
*Sphingobium arseniciresistens* Liu *et al*. 2023, 14^3^	sp. nov.	H39-3-25 (=CGMCC 1.61326=JCM 36467)	53	[3]
*Sphingomicrobium nitratireducens* You *et al*. 2022, 5^3^	sp. nov.	O-35 (=KCTC 92308=MCCC 1K07589)	25	[54]
*Sphingomonas endolithica* Tian *et al*. 2024, 4^3^	sp. nov.	ZFBP2030 (=GDMCC 1.3123=JCM 35386)^63^	18	[55]
*Sphingomonas lacusdianchii* Wang *et al*. 2024, 12^3^	sp. nov.	JXJ CY 53 (=CGMCC 1.17657=KCTC 72813)	34	[56]
*Sphingomonas pollutisoli* Liu *et al*. 2023, 13^3^	sp. nov.	H39-1-10 (=CGMCC 1.61325=JCM 36466)	53	[3]
*Staphylococcus hsinchuensis* Wang *et al*. 2024, 9^3^	sp. nov.	H164 (=BCRC 81404=NBRC 116174)	28	[57]
*Stomatohabitans* Yang *et al*. 2024, 7^3^	gen. nov.	*Stomatohabitans albus*	16	[58]
*Stomatohabitans albus* Yang *et al*. 2024, 7^3^	sp. nov.	SYSU M7M538 (=GDMCC 1.4286=KCTC 59113)^64^	16	[58]
*Stomatohabitantaceae* Yang *et al*. 2024, 7^3^	fam. nov.	*Stomatohabitans*	16	[58]
*Stomatohabitantales* Yang *et al*. 2024, 7^3^	ord. nov.	*Stomatohabitans*	16	[58]
*Streptococcus chosunensis* corrig. Lim *et al*. 2019, 1197^65^	sp. nov.	ChDC B353 (=CCUG 76564=JCM 33453)^66^	4	[59]
*Streptococcus gwangjuensis* corrig. Park *et al*. 2019, 802^67^	sp. nov.	ChDC B345 (=CCUG 76562=JCM 33299)^68^	5	[60]
*Streptococcus humanilactis* Guo *et al*. 2022, 5^3^	sp. nov.	IMAU99125 (=CCUG 77534=KCTC 21157)^69^	6	[61]
*Streptomyces camelliae* Yaxi *et al*. 2024, 5^3^	sp. nov.	HUAS 2–6 (=JCM 35918=MCCC 1K04729)^70^	41	[62]
*Streptomyces cathayae* Mo *et al*. 2024, 5^3^	sp. nov.	HUAS 5 (=JCM 36055=MCCC 1K08552)	39	[63]
*Streptomyces luomodiensis* Qi *et al*. 2024, 8^3,71^	sp. nov.	SCA4-21 (=GDMCC 4.340=JCM 36555)	67	[64]
*Stutzerimonas marianensis* (Yang *et al*. 2023) Rudra and Gupta 2024, 24^3^	comb. nov. [basonym: *Pseudomonas marianensis* Yang *et al*. 2023]	PS1 (=DSM 112238=MCCC 1 K05112)	37	[10]
*Sulfurospirillum oryzae* Xie *et al*. 2023, 6^3^	sp. nov.	SG202 (=GDMCC 1.3379=JCM 35596)	65	[65]
*Synoicihabitans* Ahmad *et al*. 2024, 9^3^	gen. nov.	*Synoicihabitans lomoniglobus*	32	[66]
*Synoicihabitans lomoniglobus* Ahmad *et al*. 2024, 9^3^	sp. nov.	LMO-M01 (=CGMCC 1.61593=KCTC 92913)	32	[66]
*Termitidicoccus* Mei *et al*. 2024, 9^3^	gen. nov.	*Termitidicoccus mucosus* ^72^	43	[67]
*Termitidicoccus mucosus* Mei *et al*. 2024, 9^3^	sp. nov.	TSB47 (=CCTCC AB 2022447=KCTC 102044)	43	[67]
*Terrimonas pollutisoli* Liu *et al*. 2024, 10^3^	sp. nov.	H1YJ31 (=CGMCC 1.61343=JCM 36215)	55	[39]
*Thermococcus sibiricus* Miroshnichenko *et al*. 2001, 90	sp. nov.	MM 739 (=DSM 12597=VKM B-2774)^73^	15	[68]
*Thermohalobaculum sediminis* (Huang *et al*. 2022) Huang *et al*. 2024, 15^3^	comb. nov. [basonym: *Limibaculum sediminis* Huang *et al*. 2022]	FT325 (=KCTC 92313=MCCC 1K07397)	36	[5]
*Tigheibacillus* Miliotis *et al*. 2024, 6^3^	gen. nov.	*Tigheibacillus jepli*	7	[38]
*Tigheibacillus halophilus* (An *et al*. 2007) Miliotis *et al*. 2024, 7^3^	comb. nov. [basonym: *Virgibacillus halophilus* An *et al*. 2007]	5B73C (=DSM 21623=JCM 21758)^74^	7	[38]
*Tigheibacillus jepli* Miliotis *et al*. 2024, 7^3^	sp. nov.	179-BFC-A-HS (=DSM 115946=NRRL B-65666)	7	[38]
*Tunturiibacter* corrig. Messyasz *et al*. 2024, 15^3,75^	gen. nov.	*Tunturiibacter lichenicola* corrig.	60	[69]
*Tunturiibacter empetritectus* corrig. Messyasz *et al*. 2024, 15^3,76^	sp. nov.	M8UP23 (=DSM 117310=HAMBI 3810)^37^	60	[69]
*Tunturiibacter gelidiferens* corrig. Messyasz *et al*. 2024, 15^3,77^	sp. nov.	M8UP39 (=DSM 117311=HAMBI 3809)^37^	60	[69]
*Tunturiibacter lichenicola* corrig. (Belova *et al*. 2018) Messyasz *et al*. 2024, 15^3,78^	comb. nov. [basonym: *Edaphobacter lichenicola* Belova *et al*. 2018]^79^	SBC68 (=DSM 104462=VKM B-3208)	60	[69]
*Tunturiibacter psychrotolerans* corrig. Messyasz *et al*. 2024, 15^3,80^	sp. nov.	X5P6 (=DSM 117309=HAMBI 3811)^37^	60	[69]
*Wagnerdoeblera faecalis* (Li *et al*. 2023) Huang *et al*. 2024, 17^3^	comb. nov. [basonym: *Falsigemmobacter faecalis* Li *et al*. 2023]	YIM 102744–1 (=CCTCC AB 2016031=KCTC 52106)	36	[5]
*Wagnerdoeblera intermedia* (Kämpfer *et al*. 2015) Huang *et al*. 2024, 17^3^	comb. nov. [basonym: *Gemmobacter intermedius* Kämpfer *et al*. 2015]	119/4 (=CCM 8510=CIP 110795=DSM 28642=LMG 28215)	36	[5]
*Xanthocytophaga* Zhang *et al*. 2023, 7^3^	gen. nov.	*Xanthocytophaga agilis*	47	[48]
*Xanthocytophaga agilis* Zhang *et al*. 2023, 7^3,81^	sp. nov.	BD1B2-1 (=GDMCC 1.2890=JCM 35374)	47	[48]
*Xanthocytophaga flava* corrig. Zhang *et al*. 2023, 8^3,82^	sp. nov.	NT2B1 (=GDMCC 1.2889=JCM 35375)	47	[48]
*Xiashengella* Huang *et al*. 2024, 7^3^	gen. nov.	*Xiashengella succiniciproducens*	52	[70]
*Xiashengella succiniciproducens* Huang *et al*. 2024, 8^3^	sp. nov.	Ai-910 (=CGMCC 1.17893=KCTC 25304)^83^	52	[70]
*Zestomonas* Rudra and Gupta 2024, 25^3^	gen. nov.	*Zestomonas thermotolerans*	37	[10]
*Zestomonas carbonaria* (Kämpfer *et al*. 2021) Rudra and Gupta 2024, 24^3^	comb. nov. [basonym: *Pseudomonas carbonaria* Kämpfer *et al*. 2021]	Wesi-4 (=CCM 9017=DSM 110367)^84^	37	[10]
*Zestomonas insulae* (Lee *et al*. 2022) Rudra and Gupta 2024, 24^3^	comb. nov. [basonym: *Pseudomonas insulae* Lee *et al*. 2022]	UL073 (=JCM 34511=KCTC 82407)	37	[10]
*Zestomonas thermotolerans* (Manaia and Moore 2002) Rudra and Gupta 2024, 24^3^	comb. nov. [basonym: *Pseudomonas thermotolerans* Manaia and Moore 2002]	CM3 (=DSM 14292=LMG 21284)	37	[10]

For references to Validation Lists 1–71, see *Int J Syst Bacteriol* **49** (1999) 1325. Lists 72–197 were published in *Int J Syst Evol Microbiol* **50** (2000) 3, 423, 949, 1415, 1699, 1953; and **51** (2001) 1, 263, 793, 1229, 1619, 1945; and **52** (2002) 3, 685, 1075, 1437, 1915; and **53** (2003) 1, 373, 627, 935, 1219, 1701; and **54** (2004) 1, 307, 631, 1005, 1425, 1909; and **55** (2005) 1, 547, 983, 1395, 1743, 2235; and **56** (2006) 1, 499, 925, 1459, 2025, 2507; and **57** (2007) 1, 433, 893, 1371, 1933, 2449; and **58** (2008) 1, 529, 1057, 1511, 1993, 2471; and **59** (2009) 1, 451, 923, 1555, 2129, 2647; and **60** (2010) 1, 469, 1009, 1477, 1985, 2509; **61** (2011) 1, 475,1011, 1499, 2025, 2563; and **62** (2012) 1, 473, 1017, 1443, 2045, 2549; and **63** (2013) 1, 797, 1577, 2365, 3131, 3931; and **64** (2014) 1, 693, 1455, 2184, 3603; and **65** (2015), 1, 741, 1112, 2017, 2777, 3767; and **66** (2016) 1, 1603, 1913, 2463, 3761, 4299; and **67** (2017) 1, 529, 1095, 2075, 3140, 4291; and **68** (2018), 1, 693, 1411, 2130, 2707, 3379; and **69** (2019), 5, 597, 1247, 1844, 2627, 3313, and **70** (2020) 1, 1443, 2960, 4043, 4844, 5596. Lists 197–218 were published in *Int J Syst Evol Microbiol* **71** (2021) 004600, 004688, 004773, 004846, 004943, 005096; and **72** (2022) 005167, 005260, 005331, 005422, 005517, 005592; and **73** (2023) 005709, 005812, 005845, 005931, 005997, 006080; and **74** (2024) 006173, 006229, 006275, 006398.

1 Abbreviations of culture collections cited in this list can be found at https://lpsn.dsmz.de/text/collections

2 Priority number assigned according to the date the documentation and request for validation are received.

3 The journal in which the name was effective published does not have continuous pages.

4 The preferred syllabification and etymology are ritt.mann’i.i. N.L. gen. n. *rittmannii*, of Rittmann.

5 The name was included in Validation List no. 84 (2002), but based on only one culture collection deposit.

6 The list editors have corrected the name to *Aliirhizobium* (A.li.i.rhi.zo’bi.um. L. masc. adj. *alius*, other; N.L. neut. n. *Rhizobium*, a bacterial genus name; N.L. neut. n. *Aliirhizobium*, another *Rhizobium*.

7 The etymology should state N.L. neut. n. and N.L. neut. adj. instead of N.L. neut. N. and N.L. neut. Adj.

8 The effective publication states that the type strain was also deposited as CECT 7176, but no documentation was supplied.

9 The effective publication states that the type strain was also deposited as CCTCC AB 2013016 and KCTC 32300, but no documentation was supplied.

10 The type species or the basonym was assigned to Risk Group 2 (German TRBA 466, “Einstufung von Prokaryonten (Bacteria und Archaea) in Risikogruppen”).

11 The effective publication states that the type strain was also deposited as ATCC 14909; CCUG 1425; CCUG 1425 A; CFBP 2437; CIP 101034; IFO 14159; JCM 5967; LMG 1224; NBRC 14159; NCCB 76044; NCTC 10367; VKM B-2171, but no documentation was supplied.

12 The authors gave TSD-286 instead of ATCC TSD-286.

13 The protologue has DSM 33098. The correct number is found in the abstract.

14 The effective publication states that the type strain was also deposited as AJ 2197; CIP 102996; DSM 6835; IAM 1568; JCM 2952; KS0036;LMG 7040; NBRC 102199, but no documentation was supplied.

15 The effective publication states that the type strain was also deposited as CCUG 51516, but no documentation was supplied.

16 The effective publication states that the type strain was also deposited as ACCC 5688 and KACC 15471, but no documentation was supplied.

17 The effective publication states that the type strain was also deposited as IAM 14884, JCM 10630, and NBRC 102411, but no documentation was supplied.

18 The effective publication states that the type strain was also deposited as CGMCC 2318, but no documentation was supplied.

19 The effective publication states that the type strain was also deposited as CECT 7516, but no documentation was supplied.

20 The effective publication states that the type strain was also deposited as CCUG 1781; CFBP 2434; CIP 75.21; IFO 14162; JCM 5966; LMG 1223; NBRC 14162; NCCB 76043; NCTC 10897; VKM B-972, but no documentation was supplied.

21 The effective publication states that the type strain was also deposited as ATCC 8062; CCUG 2087; CFBP 5589; CIP 59.11; IFO 13583; LMG 2229; NBRC 13583; NCIB 6576; NCIMB 6576; NCTC 10692; NRRL B-778; VKM B-1522, but no documentation was supplied.

22 The effective publication states that the type strain was also deposited as ATCC 17440; CCUG 51525; CFBP 2435; CIP 66.14; DSM 50188; IFO 14167; JCM 5968; NCCB 76045; NCTC 10860, but no documentation was supplied.

23 The effective publication states that the type strain was also deposited as NCIMB 14511, but no documentation was supplied.

24 The protologue states gen. nov. sp. nov. instead of sp. nov.

25 The effective publication states that the type strain was also deposited as KCTC 62951 and NBRC 113640, but no documentation was supplied.

26 The effective publication states that the type strain was also deposited as CCTCC AB 2012022 and KACC 16606, but no documentation was supplied.

27 The effective publication states that the type strain was also deposited as BCRC 80399, but no documentation was supplied.

28 The etymology should state N.L. fem. adj. *mesophila* instead of N,L. neut. adj. *mesophila*.

29 The effective publication states that the type strain was also deposited as GDMCC 62910, but no documentation was supplied.

30 The list editors have corrected *Gordonibacillus* to *Gordoniibacillus*.

31 The list editors have corrected the epithet to *rubrum* (ru’brum. L. neut. adj. *rubrum*, red).

32 The nomenclatural authority was given by the authors as Toschakov *et al.* 2015.

33 The effective publication states that the type strain was also deposited as CCTCC AB 2018346, but no documentation was supplied.

34 The effective publication states that the type strain was also deposited as MCC 3792, but no documentation was supplied.

35 The effective publication states that the type strain was also deposited as ATCC 14235; CCUG 2473; CCUG 4439; CFBP 5590; CIP 61.9; NRRL B-2649, but no documentation was supplied.

36 The syllabification and etymology should state sval.bar.den’sis. N.L. masc. adj. *svalbardensis*, pertaining to Svalbard.

37 The authors gave DSMZ instead of DSM.

38 The preferred etymology is L. gen. n. *dauci*, of the carrot.

39 Replacement name for the illegitimate name *Micromonospora ferruginea*.

40 The effective publication states that the type strain was also deposited as DSM 104294, but no documentation was supplied.

41 The effective publication states that the type strain was also deposited as KY463497, but no documentation was supplied.

42 The effective publication states that the type strain was also deposited as KCCM 43261, but no documentation was supplied.

43 The etymology should state N.L. masc. n. *Sulfitobacter* and N.L. masc. n. *Parasulfitobacter*.

44 The protologue has KCTC 72,513 instead of KCTC 72513.

45 The name of the host is *Callispongia elongata* and not *Callispongia elongate* as given in the protologue and in the title of the paper.

46 The effective publication states that the type strain was also deposited as CECT 7010, but no documentation was supplied.

47 The effective publication states that the type strain was also deposited as ATCC 51555; CCUG 49622; CFBP 5586; CIP 104204; JCM 21586; NBRC 103044; NCPPB 3063, but no documentation was supplied.

48 The effective publication states that the type strain was also deposited as CECT 8089, but no documentation was supplied.

49 The effective publication states that the type strain was also deposited as NRRL B-51283, but no documentation was supplied.

50 The effective publication states that the type strain was also deposited as ATCC 33636; CIP 106745; IAM 1598; IFO 16665; JCM 2783; NBRC 16665; NRIC 164, but no documentation was supplied.

51 The effective publication states that the type strain was also deposited as CGMCC 1.17397 and GDMCC 1.2418, but no documentation was supplied.

52 The list editors have corrected the epithet to *edaphi* (e.da’phi. Gr. neut. n. *edaphos*, soil; N.L. gen. n. *edaphi*, of soil).

53 The list editors have corrected the epithet to *scottii* (scot’ti.i. N.L. gen. n. *scottii,* named after Lewis Scott …).

54 Also validated in this list as *Mycoplana rhizolycopersici* comb. nov.

55 The effective publication states that the type strain was also deposited as ACCC 61707, but no documentation was supplied.

56 The basonym *Rhizobium rhizolycopersici* Thin *et al.* 2021 is validated in the current list.

57 The effective publication states that the type strain was also deposited as ACCC 61707, but no documentation was supplied.

58 The syllabification and etymology should state Rho.do.cy.to.pha.ga.ce’ae. N.L. fem. n. *Rhodocytophaga*, type genus of the family; -*aceae*, ending to denote a family; N.L. fem. pl. n. *Rhodocytophagaceae*, the *Rhodocytophaga* family.

59 The syllabification and etymology should state ant.arc’ti.cus. L. masc. adj. *antarcticus*, southern, of Antarctica …

60 The name was included in Validation List 80 [71], but based on only one culture collection deposit.

61 The effective publication gave as basonym *Roseibaca domitiana* Labrenz *et al.* 2024.

62 The effective publication states that the type strain was also deposited as DSM 25351 and JCM 14085, but no documentation was supplied.

63 The effective publication states that the type strain was also deposited as EE 013, but no documentation was supplied.

64 GDMCC, correctly printed in the abstract, is given in the protologue as CDMCC.

65 The list editors have corrected *chosunense* to *chosunensis* (cho.sun.en’sis. N.L. masc. adj. *chosunensis*, …).

66 The effective publication states that the type strain was also deposited as KCOM 1699, but no documentation was supplied.

67 The list editors have corrected *gwangjuense* to *gwangjuensis* (gwang.ju.en’sis. N.L. masc. adj. *gwangjuensis*, …).

68 The effective publication states that the type strain was also deposited as KCOM 1679, but no documentation was supplied.

69 The effective publication states that the type strain was also deposited as GDMCC 1.1876, but no documentation was supplied.

70 The culture collection numbers given in abstract, not in the protologue.

71 The syllabification and etymology should state N.L. masc. adj. instead of N.L. masc./fem. Adj.

72 The type species is not mentioned in the protologue, and elsewhere it was given as strain TSB47T.

73 The name was earlier validated in Validation List 82 [72], based on one culture collection deposit.

74 The effective publication states that the type strain was also deposited as KCTC 13935, but no documentation was supplied.

75 The list editors have corrected *Tunturibacter* to *Tunturiibacter* (Tun.tu.ri.i.bac’ter. N.L. masc. n. *bacter*, a rod; N.L. masc. n. *Tunturiibacter*, a rod from the tunturi, Arctic treeless fells in Finland).

76 The etymology should state N.L. part. adj. *emetritectus* instead of L. part. Adj.

77 The list editors have corrected the epithet to *gelidiferens* (ge.li.di.fe’rens. L. masc. adj. *gelidus*, ice-cold; L. pres. part. *ferens*, enduring; N.L. part. adj. *gelidiferens*, cold-enduring).

78 The protologue heading has *T.* instead of *Tunturibacter*/*Tunturiibacter*. No etymology was given in the protologue (li.che.ni’co.la. L. masc. n. *lichen*, lichen; L. masc./fem. n. suff. *–cola* (from L. masc./fem. n. *incola*), dweller, inhabitant; N.L. masc. n. *lichenicola*, inhabitant of lichens).

79 The basonym was not mentioned in the protologue.

80 The syllabification and etymology should state psy.chro.to’le.rans. Gr. masc. adj. *psychros*, cold; L. pres. part. *tolerans*, tolerating; N.L. part. adj. *psychrotolerans*, cold-enduring.

81 The etymology should state L. fem. adj.

82 The list editors have corrected the epithet to *flava* (fla’va. L. fem. adj. *flava*, yellow).

83 The protologue has KCTC 25,304 instead of KCTC 25304.

84 The effective publication states that the type strain was also deposited as CIP 111764, but no documentation was supplied.

## Corrections to earlier validation lists

The name *Robertkochia sediminum* Ma *et al*. 2022, listed in Validation List no. 209 [73] was already validated in Validation List no. 204 [74].

In Validation List no. 216 [75], the type strain of *Alsobacter ponti* Deng *et al*. 2023 was erroneously given as SYSU M600028 instead of SYSU M60028.

The type strain of *Halorubrum hochsteinianum* Vreeland *et al*. 2024, listed in Validation List no. 216 [75] was deposited as ATCC 700873 and not as ATCC 700083, the number given in the effective publication.

We thank S. Turner (NCBI, NIH, Bethesda, MD, USA) and R.R. de la Haba (University of Seville, Spain) for alerting us to the errors.
